# Identifying novel hypoxia-associated markers of chemoresistance in ovarian cancer

**DOI:** 10.1186/s12885-015-1539-8

**Published:** 2015-07-25

**Authors:** Lynda M. McEvoy, Sharon A. O’Toole, Cathy D. Spillane, Cara M. Martin, Michael F. Gallagher, Britta Stordal, Gordon Blackshields, Orla Sheils, John J. O’Leary

**Affiliations:** 1 Department of Histopathology TCD, Sir Patrick Dun’s Laboratory, Central Pathology Laboratory, St James’s Hospital, Dublin 8, Ireland; 2 Department of Obstetrics and Gynaecology, Trinity Centre for Health Sciences, St James’s Hospital, Dublin 8, Ireland; 3 Molecular Pathology Laboratory, Coombe Women and Infants’ University Hospital, Dublin 8, Ireland

**Keywords:** Hypoxia, Chemoresistance, Ovarian cancer, Cisplatin, Biomarkers

## Abstract

**Background:**

Ovarian cancer is associated with poor long-term survival due to late diagnosis and development of chemoresistance. Tumour hypoxia is associated with many features of tumour aggressiveness including increased cellular proliferation, inhibition of apoptosis, increased invasion and metastasis, and chemoresistance, mostly mediated through hypoxia-inducible factor (HIF)-1α. While HIF-1α has been associated with platinum resistance in a variety of cancers, including ovarian, relatively little is known about the importance of the duration of hypoxia. Similarly, the gene pathways activated in ovarian cancer which cause chemoresistance as a result of hypoxia are poorly understood. This study aimed to firstly investigate the effect of hypoxia duration on resistance to cisplatin in an ovarian cancer chemoresistance cell line model and to identify genes whose expression was associated with hypoxia-induced chemoresistance.

**Methods:**

Cisplatin-sensitive (A2780) and cisplatin-resistant (A2780cis) ovarian cancer cell lines were exposed to various combinations of hypoxia and/or chemotherapeutic drugs as part of a ‘hypoxia matrix’ designed to cover clinically relevant scenarios in terms of tumour hypoxia. Response to cisplatin was measured by the MTT assay. RNA was extracted from cells treated as part of the hypoxia matrix and interrogated on Affymetrix Human Gene ST 1.0 arrays. Differential gene expression analysis was performed for cells exposed to hypoxia and/or cisplatin. From this, four potential markers of chemoresistance were selected for evaluation in a cohort of ovarian tumour samples by RT-PCR.

**Results:**

Hypoxia increased resistance to cisplatin in A2780 and A2780cis cells. A plethora of genes were differentially expressed in cells exposed to hypoxia and cisplatin which could be associated with chemoresistance. In ovarian tumour samples, we found trends for upregulation of ANGPTL4 in partial responders and down-regulation in non-responders compared with responders to chemotherapy; down-regulation of HER3 in partial and non-responders compared to responders; and down-regulation of HIF-1α in non-responders compared with responders.

**Conclusion:**

This study has further characterized the relationship between hypoxia and chemoresistance in an ovarian cancer model. We have also identified many potential biomarkers of hypoxia and platinum resistance and provided an initial validation of a subset of these markers in ovarian cancer tissues.

**Electronic supplementary material:**

The online version of this article (doi:10.1186/s12885-015-1539-8) contains supplementary material, which is available to authorized users.

## Background

Ovarian cancer has recently been described as the seventh most common female cancer worldwide [1]. Moreover, it is the fifth most common cause of cancer death in women, and the leading cause of death from gynaecological malignancy in the Western world [2]. The mortality rate for ovarian cancer is quite high compared to other gynaecological cancers, mainly due to late disease presentation and the development of chemoresistance. While the majority of patients (80 %) initially respond well to chemotherapy, many patients relapse and become chemoresistant [3].

Platinum agents work by inducing intra- and inter-strand adducts in GC-rich regions of DNA [4], which in turn activate apoptosis via the p53 pathway [5]. Several mechanisms contribute to platinum resistance, including reduction in the number of copper transporters which pump the drug into the cell [6], increase in glutathione and other proteins which ‘mop up’ platinum within the cell [7], up-regulation of DNA repair mechanisms [8], and increase in the ATPase transporters which pump drug out of the cell [9].

Normal tissue oxygen tension is in the region of 4–1 %, while hypoxia is <1 % [10]; tumour hypoxia is a common feature of solid tumours, such as ovarian cancer. Several mechanisms may contribute to the development of tumour hypoxia. Rapid proliferation of tumour cells may cause depletion of available oxygen, while erratically growing tumour cells can compress blood vessels, stilting the flow of oxygenated blood to the tumour. In addition, rapid tumour growth can mean that tumour cells can grow such a distance away from blood vessels that they are beyond the diffusion distance for oxygen and can become hypoxic. Tumour hypoxia switches on genetic pathways that promote tumour aggressiveness, metastasis and chemoresistance; patients with hypoxic tumours generally have a poorer prognosis [11].

Tumour hypoxia induces activation of the hypoxia-inducible factor-1 (HIF-1) pathway. The HIF-1 protein belongs to the basic helix-loop-helix Per Ant Sim (PAS) protein family [12, 13]. It is composed of a hypoxia-regulated α subunit, and a non-hypoxia-regulated β subunit [11]. In normal oxygen, hydroxylation of proline residues within the HIF-1α oxygen dependent degradation domain targets it for proteasomal degradation via the Von Hippel-Lindau protein [11, 14]. However, in hypoxia, this does not occur; the HIF-1α protein accumulates and binds to hypoxia-regulated elements (HREs) contained within the promoter region of many genes, such as those that regulate metabolism, cell survival, angiogenesis and invasion [11].

Hypoxia induces resistance to a wide range of cytotoxic agents in a number of different cancer types including ovarian cancer [15]. Hypoxia has been shown to induce platinum resistance through interference with a number of biological molecules such as L1-cell adhesion molecule (L1-CAM) [16], signal transducer and activator of transcription 3 (STAT3) [15] and p53 [17]. The presence of hypoxia, measured by tumour expression of HIF-1α or surrogate markers of hypoxia such as glucose transporter (GLUT)-1 or carbonic anhydrase 9 (CA9), has been shown to be associated with poorer survival in ovarian cancer patients [18, 19]. However, the correlation of HIF-1α with clinical response is complex; increased HIF-1α expression has also been linked to improved survival [20].

While hypoxia has been previously shown to induce chemoresistance in a number of cell line models, few studies evaluate the influence of hypoxia on platinum resistance in ovarian cancer. In addition, although several previous studies have explored links between ovarian cancer genes and hypoxia [21–23], to our knowledge there is no published study which has carried out whole-genome profiling of ovarian cancer cell lines that have been exposed to hypoxia in combination with cytotoxic chemotherapy. Furthermore, although HIF-1α is frequently cited as a marker of hypoxia, its role in predicting clinical response to hypoxia is unclear.

As hypoxia is being progressively revealed as an important factor in the development of chemoresistance, it is important to discover new hypoxia-associated biomarkers which may be exploited for their prognostic and therapeutic potential in ovarian cancer. In order to explore the relationship between platinum resistance and hypoxia, we selected a paired cisplatin resistance ovarian cancer cell line model (A2780/A2780cis) [24–26]. We developed a ‘hypoxia exposure matrix’ which was based on potential clinical scenarios and exposed cells to hypoxia prior to and during treatment with cisplatin and measured relative changes in platinum resistance compared to cells treated in normal oxygen conditions via the 3-(4,5-dimethylthiazol-2-yl)-2,5-diphenyltetrazolium bromide (MTT) assay. We then took cells that had been exposed to concomitant hypoxia and/or platinum (cisplatin) without any prior exposure to hypoxia and carried out whole genome profiling using Affymetrix Human Gene 1.0 ST arrays. Following pathway analysis of genes differentially expressed following exposure to hypoxia/platinum, we selected genes which had been linked to platinum resistance in the literature. We examined their expression in a cohort of serous papillary ovarian tumour samples grouped according to response to chemotherapy using reverse transcription polymerase chain reaction (RT-PCR).

## Methods

### Cell culture

The human epithelial serous ovarian cancer cell lines A2780 (cisplatin-sensitive) and A2780cis (cisplatin-resistant) were purchased from the European Collection of Cell Cultures (ECACC, UK) and cultured in a humidified atmosphere at 37 °C, 5 % CO_2_. They were maintained in RPMI 1640 medium (Sigma, UK) supplemented with 10 % foetal bovine serum (FBS, Lonza, UK), 1 % penicillin/streptomycin mixture (Lonza, UK) and 2 mM Glutamax (Gibco, Biosciences, Ireland). In addition, A2780cis were cultured in 1 μM cisplatin (Hospira, UK) every second passage in accordance with the ECACC guidelines. Cells were regularly checked for signs of bacterial, fungal or mycoplasmal contamination.

### Tumour samples

Paraffin embedded sections were cut from 35 tumour specimens obtained following surgery for ovarian cancer. Patients provided written informed consent for their samples to be used and ethical approval was obtained from the St James’s Hospital/the Adelaide and Meath Hospital, Dublin incorporating the National Children’s Hospital Research Ethics Committee, Dublin, Ireland. The study was carried out in accordance with the principles of the Declaration of Helsinki. Samples were divided into responders, partial responders, and non-responders based on their response to platinum/taxane-based chemotherapy (Table 1).

**Table 1:** Clinical characteristics of tumour samples^a^ based on response to chemotherapy

Class	Definition	Stage/Grade	Number of Samples
Responders	Recurrence >12 months following completion of chemotherapy	4/3	1
		4/2	1
		3/3	4
		3/2	5
		2/3	2
		1/3	2
		1/-	1
		Total	16
Partial Responders	Recurrence between 6 – 12 months following completion of chemotherapy	4/-	1
		3/3	5
		3/2	2
		3/-	1
		-/3	1
		−/−	1
		Total	11^b^
Non-Responders	Recurrence <6 months following completion of chemotherapy	4/3	1
		3/3	3
		3/2	2
		-/2	1
		Total	7

^a^All tumours were serous adenocarcinomas. Patients who were classed as between two stages/grades are included in this table as the higher stage/grade

^b^This group included one recurrent primary peritoneal serous adenocarcinoma

-, no information available

### Drug treatment

Cells were treated with cisplatin (Hospira, UK) that was kindly donated by the compounding unit at St. James’s Hospital, Dublin. It was received as a 1 mg/ml solution and was freshly diluted in media to the desired stock concentration directly before each experiment. A vehicle control consisting of 1 mg/ml mannitol (Sigma, UK) and 9 mg/ml sodium chloride (Sigma, UK) was also freshly prepared in media prior to each experiment. All drug treatments were for 3 days.

### Hypoxic exposure

**Fig. 1:**
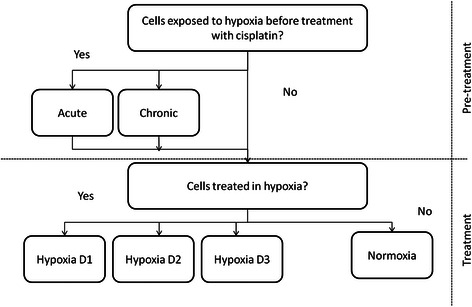
Hypoxia matrix treatment pathway. In the pre-treatment phase, cells received no, acute or chronic hypoxia. During treatment, cells that had prior hypoxia exposure were either treated in normoxia or hypoxia. Cells that had no prior hypoxia exposure were treated in normoxia for the full 3-day treatment period or in hypoxia for 1, 2 or 3 days, with any remaining treatment time in normoxia

Hypoxia (0.5 % O_2_, 5 % CO_2_) was achieved using the INVIVO2 400 hypoxia workstation (Ruskinn, UK). For the MTT assay experiments, cells were cultured in 96-well microtitre plates (Sarstedt, Germany) at a concentration of 5,000 cells/well for various time periods as part of a hypoxia design matrix (Table 2). The matrix consisted of two phases – pre-treatment (up to 5 days) and treatment (3 days) (Fig. 1, Table 2). In the pre-treatment phase, cells were either maintained in normal oxygen (normoxia) or exposed to hypoxia for an acute (4 h) or chronic (5 days) time period. During the treatment phase, the cells were treated with cisplatin in either normoxia or hypoxia. Cells that had had been exposed to hypoxia prior to drug treatment were treated with cisplatin in either normoxia or hypoxia for the entire duration of treatment. The effect of introduction of hypoxia during drug treatment was investigated by challenging hypoxia naïve cells (i.e. cells with no pre-exposure to hypoxia) with concurrent cisplatin and hypoxia, either for the full treatment period, or for part of the treatment period (1 or 2 days, with the remainder in normoxia). For RNA and protein extraction, cells were cultured in T75 tissue culture flasks (Sarstedt, Germany) at a concentration of 3–6 × 10^4^/cm^2^ (A2780) or 1 × 10^3^–1 × 10^4^/cm^2^ (A2780cis) as per the ECACC guidelines. For drug treatments, cells were briefly removed from the hypoxia chamber and brought to the laminar flow hood. Following drug treatment, the cells were immediately returned to the hypoxia chamber.

**Table 2:** Design matrix of hypoxia/cisplatin treatments

Pre-treatment	Treatment Day 1	Treatment Day 2	Treatment Day 3
	(0 – 24 h)	(24 – 48 h)	(48 – 72 h)
Normoxia	Normoxia	Normoxia	Normoxia
Normoxia	Hypoxia	Hypoxia	Hypoxia
Normoxia	Normoxia	Hypoxia	Hypoxia
Normoxia	Normoxia	Normoxia	Hypoxia
4 hours hypoxia	Normoxia	Normoxia	Normoxia
4 hours hypoxia	Hypoxia	Hypoxia	Hypoxia
5 days hypoxia	Normoxia	Normoxia	Normoxia
5 days hypoxia	Hypoxia	Hypoxia	Hypoxia

### MTT Assay

Initial experiments were carried out to determine the appropriate seeding density and drug treatment length. Cells were plated in 100 μL of medium and left overnight to attach. The following morning, they were treated with cisplatin at varying concentrations for 3 days. Following treatment, response was measured via an MTT assay carried out in accordance with the manufacturer’s instructions (Roche, UK). Absorbance was read at 570 nm on an optical plate reader (Dynex Technologies, US). The absorbance detected was directly proportional to the number of live cells present.

### Protein preparation and quantification

A timecourse of HIF-1α protein expression was carried out to monitor its levels during hypoxic exposure. Protein was extracted in normoxia, and following 4 h, 3 days and 5 days of exposure to hypoxia. Cells were scraped into ice-cold phosphate buffered saline (PBS, Lonza, UK), spun down and lysed with ice-cold radioimmunoprecipitation (RIPA) buffer (Sigma, UK) supplemented with 1 % protease inhibitor (Sigma, UK), 1 % phosphatase inhibitor (Sigma, UK) and 2 mM phenylmethanesulfonylfluroride (PMSF, Sigma, UK). For hypoxic protein extractions, PBS and lysis buffer were allowed to equilibrate in the hypoxia chamber, removed in a sealed container shortly before extraction, and placed on ice. Protein was prepared from hypoxic samples within the hypoxia chamber. A positive control for HIF-1α expression was prepared by exposing A2780 cells to 50 μM CoCl_2_, a hypoxia mimetic which stabilizes HIF-1α protein in normal oxygen, for 24 h. Protein was quantified with the bicinchoninic acid (BCA) assay; samples were stored at −20 °C.

### Sodium dodecyl sulphate-polyacrylamide gel electrophoresis (SDS-PAGE) and western blotting

The prepared protein (30 μg) was electrophoresed on 12 % gels; wet transfer was used to transfer protein from the gels to nitrocellulose membranes (Biorad, Ireland). The membranes were blocked with 5 % skimmed milk in PBS (Oxoid, Thermofisher, Denmark) 0.1 % Tween (Sigma, UK) for 2 h on an orbital shaker (Stuart Scientific, UK) at 4 °C, then probed with mouse α-HIF-1α (1:250, Clone 54, BD Biosciences, UK) in 3 % skimmed milk overnight at 4 °C. The following day the membranes were washed in PBS 0.3 % Tween, and probed with horse radish peroxidise (HRP)-conjugated α-mouse (1:1,000, Cat. A6782, Sigma, UK) for 1 h at room temperature. Blots were incubated with Amersham ECL Advance (GE Healthcare, UK) for 1 min and chemiluminescent images were acquired using a Fuji Luminescent image analyzer LAS-4000. The probes were then blocked again for 2 h at 4 °C and incubated with β-actin (1:10,000, Cat. A5441 Sigma, UK) overnight at 4 °C. The following day, the blots were washed in PBS 0.3 % Tween and probed with AP-conjugated α-mouse (1:1,000, Cat. A4312, Sigma, UK).

### RNA Extraction

RNA was extracted from cell lines using the RNeasy Mini kit (Qiagen, UK) according to the manufacturer’s instructions. Eluted RNA was stored at −80 °C. RNA yield was assessed using the NanoDrop (Thermofisher, Denmark) and RNA quality was determined using the Bioanalyzer (Agilent, US). RNA was extracted from formalin fixed paraffin embedded (FFPE) ovarian tumour samples using the RNeasy FFPE kit (Qiagen, UK) according to the manufacturer’s instructions. Sections were stained with haematoxylin and eosin and pathologically reviewed; if tumour cell density was >90 %, whole sections were used for extraction. If a significant stromal component was present, the sections were macrodissected to enrich for the epithelial tumour population.

### Affymetrix array analysis

RNA was extracted from cells treated with cisplatin for 3 days in the presence and absence of hypoxia and interrogated on microarrays. Three independent biological replicates were interrogated for each condition:

- A2780 (normoxia, untreated)
- A2780 (hypoxia, untreated)
- A2780 (normoxia, cisplatin treated)
- A2780 (hypoxia, cisplatin treated)
- A2780cis (normoxia, untreated)
- A2780cis (hypoxia, untreated)
- A2780cis (normoxia, cisplatin treated)
- A2780cis (hypoxia, cisplatin treated)

In total, 24 arrays were carried out. All samples run on the arrays had an RNA Integrity Number (RIN) > 9.5 (Bioanalyzer, Agilent, USA), indicating that the RNA was of high quality. Samples were prepared according to the manufacturer’s instructions. Quality control metrics were carried out based on the Affymetrix quality control white paper [27]. Data was analysed using the Bioconductor libraries ‘oligo’, ‘limma’ and ‘made4’ [28–30]. Data was normalized using the robust multi array average (RMA) method [31] and statistical differences in gene expression across arrays was determined using limma. A fold change ≥2 and false discovery rate (FDR) < 0.05 was determined as significant. Pathway analysis was carried out on lists of genes which were determined as significant using DAVID v6.7 [32, 33]. Individual gene function and interaction was determined using PubMed and the online tool information hyperlinked over proteins (iHOP) [34]. Microarray data are available in the ArrayExpress database (www.ebi.ac.uk/arrayexpress) under accession number E-MTAB-3645.

### Taqman PCR

Expression of potential markers of chemoresistance in ovarian cancer selected following analysis of gene lists from the Affymetrix analysis was determined using Taqman PCR in a cohort of ovarian tumour samples. RNA was extracted from 35 serous adenocarcinomas of mixed stage and grade (Table 1). cDNA was created using the High Capacity RNA-to-cDNA kit (ABI, USA) and Taqman PCR was carried out using Applied Biosystems Universal Master Mix II (without UNG) and Gene Expression Assays. Gene expression was determined for ANGPTL4, HER3 (ERBB3) and HIF-1α. Glyceraldehyde 3-phosphate dehydrogenase (GAPDH) was used as an endogenous control. Relative gene expression was determined using the comparative C_T_ method (2^-ΔΔCT^) [35].

### Statistical analyses

All experiments were carried out for n = 3. For MTT assay experiments, response to cisplatin was measured by changes in the inhibitory concentration 50 (the concentration of drug required to kill 50 % of cells, IC_50_). Results were plotted using GraphPad Prism Software, Version 5.03 (GraphPad Software Inc., USA). Non-linear regression was used to analyse the growth curves. 100 % was set as the average absorbance of untreated cells, and all other points on the graph were calculated with the following equation:

$$ \%\;\mathrm{Survival}=\frac{\mathrm{Absorbance}\ \mathrm{of}\ \mathrm{Treated}\ \mathrm{Cells}}{\mathrm{Absorbance}\ \mathrm{of}\ \mathrm{Untreated}\ \mathrm{Cells}}\times 100 $$

Student’s t-tests on the IC_50_ values were used to compare the IC_50_ values at different points of the matrix. Significance was set at p < 0.05.

For the microarray analysis, Limma was used to determine significant differences in gene expression. Significance was set at a fold-change ≥ 2, with an FDR < 0.05. For Taqman analysis of changes in gene expression, fold changes in gene expression were calculated using the 2^-ΔΔCT^ method. Individual fold-changes for each of the responder samples were calculated by subtracting the ΔC_T_ (gene expression C_T_ normalised to the endogenous control, GAPDH) for each sample from the average ΔC_T_ for the group to obtain ΔΔC_T_ and was entered into the formula 2^-ΔΔCT^ to obtain the fold change in order to evaluate the variance among the responders. In partial and non-responders, ΔC_T_ was obtained by subtracting the ΔC_T_ for each sample from the average ΔC_T_ for responders to obtain ΔΔC_T._ Unpaired two-sample t-tests were carried out on the fold changes for partial and non-responders vs responders to determine significant changes in gene expression, *p* < 0.05.

## Results

### Acute hypoxia induces resistance to cisplatin in A2780 and A2780cis

**Fig. 2:**
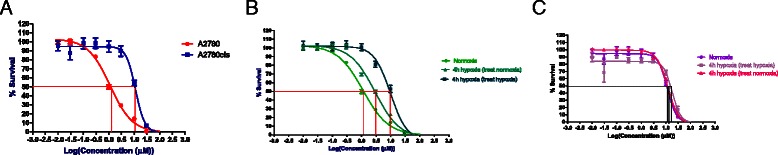
Response of A2780 and A2780cis to Cisplatin following Acute Hypoxia. **a**. A2780cis were 9-fold more resistant to cisplatin in normal oxygen. **b**. Following acute hypoxia, A2780 cells were 8-fold more resistant to cisplatin if the treatment was also carried out in hypoxia. This was attenuated (2.4-fold) if the treatment was carried out in normoxia. **c**. A2780cis were approximately 2-fold more resistant to cisplatin following acute hypoxia if the treatment was carried out in hypoxia. When acutely hypoxic A2780cis were placed in normal oxygen for the treatment period, the resistance returned to the same level as cells which were never exposed to hypoxia. *n* = 3 *p < 0.05 ***p < 0.001

A2780cis had a significantly higher IC_50_ for cisplatin than A2870, *p* < 0.001 (Fig. 2a). In A2780 cells, exposure to hypoxia for 4 h before treatment, followed by treatment with cisplatin in hypoxia resulted in an 8-fold increase in IC_50_ compared with normoxic cells (Fig. 2b), *p* < 0.001. If the acutely hypoxic A2780 cells were treated with cisplatin in normoxia, the resistance was reduced to 2.4-fold, however, this was still significant when compared to the normoxic cells (*p* < 0.01). A2780cis cells that were exposed to acute hypoxia prior to treatment in hypoxia displayed a further 2-fold increase in resistance to cisplatin which was significant *p* < 0.05 (Fig. 2c). However, if cells were removed from hypoxia for the treatment period, the resistance level was equivalent with that of normoxic cells.

### Chronic hypoxia induces resistance to cisplatin in A2780 and A2780cis

**Fig. 3:**
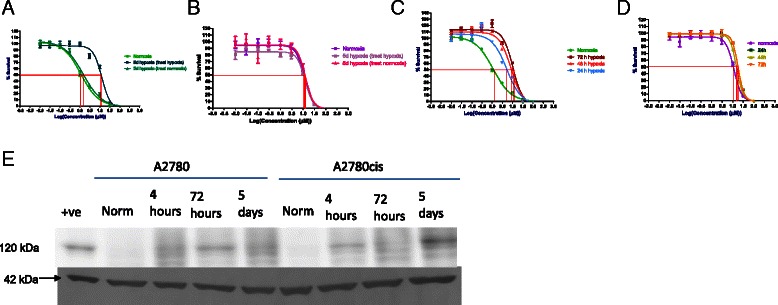
Response of A2780 and A2780cis to chronic hypoxia and hypoxia during treatment. **a**. A2780 cells exposed to chronic hypoxia before treatment with cisplatin resulted in a 10-fold increase in resistance when the treatment was also carried out in hypoxia. The resistance returned to that of normoxia when the chronically hypoxic cells were returned to normal oxygen for the treatment period. **b**. A2780cis displayed more modest changes in resistance (<2-fold) following hypoxia although this was still significant. **c**. Hypoxia naïve cells (cells not exposed to hypoxia before treatment) were exposed to hypoxia during cisplatin treatment for all or part of the 72 h treatment period for A2780. **d**. Hypoxia naïve cells (cells not exposed to hypoxia before treatment) were exposed to hypoxia during cisplatin treatment for all or part of the 72 h treatment period for and A2780cis. Both cell lines developed resistance when cells were challenged with cisplatin and hypoxia at the same time without any previous exposure, although the fold changes were more modest in A2780cis. **e**. Timecourse of HIF-1α protein expression (120 kDa). Loading control β-actin also shown (42 kDa). HIF-1α protein was absent in normoxia in A2780 and A2780cis. The levels fluctuated slightly over time, with an increase in HIF-1α expression at 3 days in A2780 and a decrease in HIF-1α expression in A2780cis at 3 days. A2780 cells were exposed to 50 μM CoCl_2_ for 24 h for a positive control. *n* = 3 *p < 0.05 **p < 0.01 ***p < 0.001

Pre-exposing A2780 cells to chronic hypoxia (5 days) followed by treatment with cisplatin in hypoxia resulted in almost a 10-fold increase in IC_50_ (Fig. 3a) (*p* < 0.001). Cells that were chronically exposed to hypoxia but treated with cisplatin in normoxia showed comparable sensitivity to hypoxia as non-hypoxic cells. Pre-exposing A2780cis cells to hypoxia for 5 days before treatment with cisplatin in hypoxia resulted in a 10 % increase in resistance (Fig. 3b) (*p* < 0.05). This increase was only statistically significant when cells were both pre-exposed to hypoxia and treated with cisplatin in hypoxia.

### Treating A2780 and A2780cis in hypoxia increases resistance to cisplatin

A2780 cells which were grown in normoxia and treated in hypoxia (hypoxia naïve cells) showed increased resistance to cisplatin (Fig. 3c). Cells which had the full 3-day treatment in hypoxia showed levels of resistance which were comparable with the resistance seen in the cells which had been chronically exposed to hypoxia prior to drug treatment in hypoxia. The level of resistance in A2780 cells increased with increasing length of time in hypoxia during the drug treatment. Hypoxia naïve A2780cis cells which were treated with cisplatin in hypoxia also demonstrated increased resistance to cisplatin (Fig. 3d).

### Patterns of HIF-1α protein expression in hypoxia in A2780 and A2780cis

HIF-1α protein was undetectable in both cell lines in normal oxygen conditions (Fig. 3e). However, protein was expressed from 4 h hypoxia exposure in both cell lines. Levels of HIF-1α fluctuated slightly over time, with an increase in HIF-1α protein expression observed at 3 days in A2780, but a decrease in HIF-1α protein expression observed in A2780cis.

### Whole genome comparison of A2780 and A2780cis

In total, 1202 genes were differentially expressed in A2780cis compared to A2780. Of these, 511 were up-regulated and 691 were down-regulated. Gene expression changes are graphically represented on heat map and chromosomal location plot (Additional file 1: Figure S1A, D). Pathway analysis on Database for Annotation Visualization and Individual Discovery (DAVID) revealed the top up-regulated pathways as gap junction, cancer pathways and intra-cellular signalling (Table 3), while top down-regulated pathways include adhesion pathways (Table 4).

**Table 3:** Significantly up-regulated pathways in A2780cis compared to A2780

Pathway	Genes	P-value
Gap Junction	GNAI1, GUCY1A3, GUCY1B3,ITPR3, PDGFC, PDGFA, PrKCA, PrKCB, TUBB4	0.005
Pathways in Cancer	Fas, Jak1, KITLG, AR, ARNT2, CTNNA3, FGF1, FGF10, FGFR2, ITGA6, Jun, PPARγ, PLD1, VEGFC	0.01
Calcium Signalling	ATP2B4, CHRNA7, CACNA1H, CAMK4, CYSLTR2, GNAL, PTGER3, P2RX5, ERBB3	0.02
PPAR Signalling	CD36, ACSL1, CPT1A, FABP5, MMP1, SLC27a2	0.02
Long-term depression	PLA2G3	0.02

**Table 4:** Significantly down-regulated pathways in A2780cis compared to A2780

Pathway	Genes	P-value
Focal Adhesion	FYN, SHC4, ACTN3, CAV1, CAV2, COL1A2, COL6A3, FLNC, HGF, IGF1R, ITGA5, ITGA8, LAMA1, PIK3CA, PDGFD, PDGFA, SPP1, THBS1, AKT3, VAV3, VCL	<0.0001
Arrhythmogenic Right Ventricular Cardiomyopathy	CDH2, CACNG7, DSC2, DSG2, DMD, CACNA1C, JUP, SLC8a1, TCF7L1	<0.0001
Melanoma	CDKN2A, FGF18, FGF20, FGF5	0.001
Axon Guidance	EPHA3, EPHA7, NTNG1, PLXNC1, ROBO2, SEMA3E, SEMA6A, SEMA6D, SLIT2, UNC5C	0.006
Cell Adhesion Molecules	CDH2, CLDN17, CLDN8, CNTNAPA2, HLA-DPA1, HLA-DRB3, NEO1, NLGN4X, NEGR1, SDC2, VCAN	0.006

### Hypoxia induces common pathways in A2780 and A2780cis

In A2780 and A2780cis, 914 genes were commonly altered in response to treatment with cisplatin in hypoxia. Chromosomal location plots display the location of alterations in gene expression while heat maps graphically represent the differential gene expression (Additional file 1: Figure S1B, C, E, F). Similar pathways were altered in both cell lines in response to hypoxia. In both cell lines, the top up-regulated pathways included focal adhesion and mitogen activated protein (MAP) kinase signalling, while the top down-regulated pathways included DNA replication, cell cycle and base excision repair (Table 5). We found down-regulation of cell cycle molecules including CDC25A, DNA replication genes including the minichromosome maintenance proteins (MCMs) and pyrimidine metabolism genes in both cell lines when exposed to hypoxia. When A2780 cells exposed to hypoxia (hypoxia-induced resistance) were compared with A2780cis cells (A2780 cells exposed to repeated cisplatin exposure, cisplatin-induced resistance), 128 genes were commonly altered. From this commonly altered gene list, the MAP kinase signalling pathway was again found to be significantly enriched, while DNA replication was down-regulated.

**Table 5:** Pathway analysis of genetic changes in A2780 and A2780cis in response to hypoxia

Cell Line	Pathway	P-value	Change in Expression
A2780	MAPK signalling	0.001	Up-Regulated
	Focal adhesion	0.002	
	Renal Cell Carcinoma	0.01	
	Starch and Sucrose Metabolism	0.04	
	Complement and Coagulation Cascade	0.04	
A2780cis	MAPK signalling	0.02	
	Focal adhesion	<0.001	
	Axon guidance	0.002	
	TGF beta signalling	0.007	
	Toll like receptor signalling	0.02	
	DNA replication	<0.001	
A2780	Cell cycle	<0.001	Down-regulated
	Pyrimidine metabolism	<0.001	
	Base Excision Repair	<0.001	
	Homologous Recombination	<0.001	
	DNA replication	<0.001	
A2780cis	Cell cycle	<0.001	
	Pyrimidine metabolism	0.001	
	Base excision repair	0.004	
	Oxidative phosphorylation	<0.001	

### Genetic alterations in the ‘hypoxic Only’ response to cisplatin

The gene expression differences in A2780 and A2780cis in response to cisplatin were compared in normoxia and hypoxia. We then looked at the ‘hypoxic only’ response i.e. the genes which were altered in response to cisplatin in hypoxia but not normoxia, as these may account for some of the increased resistance to cisplatin observed in hypoxia. Pathway analysis of these genes in A2780 revealed that there was up-regulation of apoptotic pathways, ATP-binding cassette (ABC) transporters and cancer pathways, while in A2780cis there was up-regulation of the focal adhesion pathway. In both cell lines there was down-regulation of the systemic lupus erythematosus pathway, containing histone encoding genes as well as down-regulation of cell cycle and erbb signalling (A2780) and homologous recombination and amino acid degradation pathways (A2780cis) (Table 6).

**Table 6:** Pathway analysis of genetic changes in the ‘hypoxic only’ response to cisplatin in A2780 and A2780cis

Cell Line	Pathway	P-value	Change in Expression
A2780	Apoptosis	0.001	Up-regulated
	ABC Transporters	0.002	
	Amyotrophic Lateral Sclerosis	0.005	
	Small Cell Lung Cancer	0.02	
	p53 signalling	0.02	
	Pancreatic Cancer	0.02	
	Chronic Myeloid Leukemia	0.03	
A2780cis	Focal Adhesion	0.04	
A2780	Systemic Lupus Erythematosus	0.02	Down-regulated
	MAPK signalling	<0.001	
	Cell Cycle	0.002	
	Steroid Biosynthesis	0.005	
	ErbB Signalling	0.01	
	Nitrogen Metabolism	0.02	
	Axon Guidance	0.02	
	Colorectal Cancer	0.03	
	Gap Junction	0.04	
A2780cis	Systemic Lupus Erythematosus	<0.001	
	Valine, Leucine and Isoleucine degradation	0.004	
	Homologous Recombination	0.03	
	Oocyte meiosis	0.05	

### Hypoxia-associated biomarker selection

Potential novel biomarkers of hypoxia in ovarian cancer were identified from the gene lists generated by Affymetrix analysis by thorough literature searching of their expression and significance in ovarian cancer and others. Two potentially novel biomarkers of hypoxia in ovarian cancer were selected – angiopoietin like protein 4 (ANGPTL4, up-regulated in both A2780 and A2780cis in response to hypoxia exposure) and human epidermal growth factor receptor 3 (HER3, up-regulated in A2780cis compared to A2780, and in hypoxic A2780 compared to normoxic A2780 while down-regulated in the A2780cis ‘hypoxic only’ response to cisplatin.

### Biomarker expression in serous papillary adenocarcinoma

**Fig. 4:**
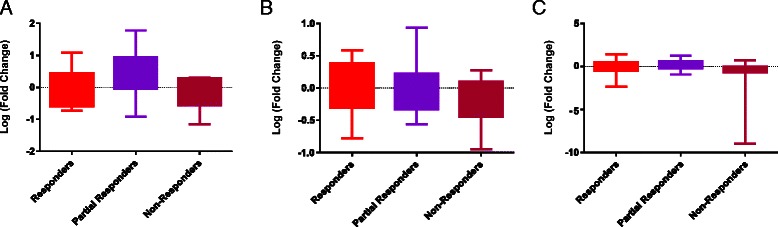
Expression of potential and known hypoxia biomarkers in ovarian cancer samples. The expression of ANGPTL4 (**a**), HER3 (**b**) and HIF1α (**c**) was examined in 35 serous ovarian adenocarcinoma samples. The samples were divided into responders (*n* = 16), partial responders (*n* = 11) and non-responders (*n* = 8). Expression of ANGPTL4 trended towards up-regulation in partial and down-regulation in non-responders compared to responders to chemotherapy. Expression of HER3 trended towards down-regulation in partial and non-responders to chemotherapy compared to responders. Expression of HIF-1α trended towards down-regulation in non-responders compared to responders to chemotherapy. There were missing data in one patient for HER3 expression and in three patients for ANGPTL4 expression in the responder group; and in one patient for ANGPTL4 expression in the non-responder group

Expression of ANGPTL4, HER3 and HIF-1α was examined in 35 serous papillary carcinomas. The patient/tumour characteristics are described in Table 1. ANGPTL4 demonstrated a trend for up-regulation in partial responders and down-regulation in non-responders to chemotherapy compared to responders (Fig. 4a). HER3 trended towards down-regulation in both partial and non-responders to chemotherapy compared to responders (Fig. 4b), while HIF-1α appeared unchanged in partial responders and trended towards down-regulation in non-responders to chemotherapy compared to responders (Fig. 4c).

## Discussion

We developed a hypoxia matrix in order to best represent possible clinical scenarios in patient care. We considered four types of patient:

- A patient with a small tumour which has not been hypoxic before or during chemotherapy (normoxia)
- A patient with a large tumour receiving neoadjuvant chemotherapy before debulking surgery (pre-exposure to acute/chronic hypoxia, treatment in hypoxia)
- A patient with a large tumour removed before receiving adjuvant chemotherapy (pre-exposure to acute/chronic hypoxia, treatment in normoxia)
- A patient with a tumour undergoing transient hypoxia due to abnormal vasculature and compression of blood vessels (all matrix conditions that included exposure to hypoxia)

Exposing both A2780 and A2780cis to acute or chronic hypoxia before treating with cisplatin increased resistance to cisplatin, but only if the treatment period was also carried out in hypoxia. This indicated that the resistance which could be induced by hypoxia was quickly reversible when the cells were moved back into normal oxygen. Reoxygenation following hypoxic exposure has been shown to restore sensitivity to radiation therapy in breast cancer [36] and gastric cancer [37], and a previous study in breast cancer has shown that cells exposed to hypoxia followed by drug treatment in normal oxygen displayed no resistance to cisplatin [38].

We found that by exposing the cells to hypoxia during treatment without any prior hypoxia exposure, the cells had similar resistance levels to those which had undergone chronic pre-exposure to hypoxia, indicating that the most important factor determining chemoresistance is the presence of hypoxia at the time of treatment, rather than prior exposure. This indicates a potential role for hypoxia-targeted agents in combination with standard chemotherapy regimens. This may be particularly important in patients undergoing neoadjuvant chemotherapy, as these patients often have large tumours that may be hypoxic.

The mechanisms underlying the platinum resistance observed in A2780cis have not been fully identified. Upregulation of cell proliferation markers [39], members of the Akt signalling pathway [40, 41], DNA-repair mechanisms [26] and ATP-dependent processes [42], and reduction in the copper transporter CTR1, thus preventing platinum accumulation [43] are among the suggested influences. We compared A2780 and A2780cis at the whole genome level in order to further identify mechanisms by which A2780cis has become resistant to cisplatin. Large differences in gene expression were observed as shown in the heat map (Additional file 1: Figure S1). We found up-regulation of p53 pathway signalling, a pathway that has been implicated in response to DNA damage [44]. In addition, we found down-regulation of other gene families linked to chemoresistance including several of the ATP-ase transporters and glutathione peroxidise 8. Knockdown of glutathione peroxidise 3 has been shown to increase platinum sensitivity in ovarian cancer clear cell carcinoma [45]. Other genes identified in the current study which may contribute to the cisplatin resistance of A2780cis included up-regulation of PDGF isoforms such as PDGFC which has been previously linked to cisplatin resistance in head and neck squamous cell carcinoma cell lines [46]. Janus kinase (Jak) 1, the tyrosine kinase protein which has been linked to cisplatin resistance in breast carcinoma [47] and ovarian cancer [48] was also up-regulated in A2780cis. Caveolin 1 (CAV1), was found to be down-regulated in A2780cis. Low CAV1 expression has been linked to cisplatin resistance in oral squamous cell carcinoma [49] while it is a putative tumour suppressor candidate in ovarian cancer [50].

We found that common pathways were significantly enriched in both cell lines in response to hypoxia, although not necessarily the same genes. Far more genes were down-regulated in A2780 cells in response to hypoxia compared with A2780cis as can be seen on the chromosomal location plots (Additional file 1: Figure S1). Genes linked to cellular proliferation were markedly down-regulated in both cell lines. Chemotherapy drugs generally target actively dividing cells and reduced cell proliferation has been suggested as a mechanism of chemoresistance [51]. We found reduced expression of cell cycle markers, DNA replication markers and metabolic markers in both A2780 and A2780cis cells that were exposed to hypoxia, which may have contributed to the resistance observed. Low cellular proliferation has been linked to chemoresistance in clear cell carcinoma of the ovary [52, 53]. Despite being amenable to surgical excision, low grade ovarian serous tumours (characterised by a low mitotic index) have been shown to be relatively resistant to chemotherapy [54]. Approximately 1,000 fewer genes altered in hypoxia in A2780cis compared to A2780, suggesting that some of the changes induced by hypoxia in A2780 may have already been induced in A2780cis through cisplatin exposure. In addition, 128 genes which were altered by hypoxia in A2780 were already changed in A2780cis.

We did not see any change in HIF-1α expression in response to hypoxia at the gene level in the arrays, however, HIF-1α is known to be regulated at the protein level [55] and we had found that HIF-1α protein was increased in response to hypoxia in these cell lines. In addition, we did see up-regulation of surrogate hypoxic markers in hypoxia-exposed cells such as GLUT-1 (2.61-fold in A2780) and CA9 (20.42-fold in A2780 and 4.18-fold in A2780cis). We identified many genes altered in response to hypoxia in our cell line model which have been previously linked to platinum resistance in the literature including complement decay accelerating factor (CD55) [56] and tissue inhibitor of metallopeptidases 3 (TIMP3) [57]. We looked at the differences in genetic response to cisplatin in normoxia and hypoxia in both cell lines, and mainly focused on the changes which occurred in hypoxia which did not occur in normoxia, as these are likely linked to the platinum resistance which occurred when the cells were exposed to hypoxia. We found many potential biomarkers of platinum resistance in hypoxia including NOTCH1 [58] which has been identified as a potential therapeutic target in ovarian cancer [59, 60].

We carried out a comprehensive literature search of the genes which we identified on our array analysis in order to identify markers which could serve as novel markers of hypoxia and/or platinum resistance in ovarian cancer. We chose ANGPTL4 and HER3 to follow up in a cohort of ovarian tumour samples. Previous studies [61–64] have indicated a negative role for ANGPTL4 in other cancer types, and ANGPTL4 has previously been shown to be activated by HIF-1α [65] and to confer protection against hypoxia-induced apoptosis in cell lines. However, there was little information available regarding its role in ovarian cancer and platinum resistance. HER3 has previously been identified as a potential therapeutic target in ovarian cancer and has been linked to sensitivity to monoclonal antibody therapy with gefitinib [66] and pertuzumab [67]. Gastric adenocarcinoma cells knocked down for HER3 have shown increased sensitivity to cisplatin [68]. However, little is known about the influence of hypoxia on HER3 expression. In addition, we evaluated HIF-1α expression, a universal marker of hypoxia.

ANGPTL4 is an angiogenesis-associated protein which has many functions including prevention of apoptosis [69], induction of angiogenesis [70], inhibition of angiogenesis [71] and facilitation of metastasis [72]. We found it to trend towards up-regulation in partial responders to chemotherapy compared to responders. This was a novel finding, as there is no information in the literature regarding ANGPTL4 expression in serous ovarian cancer. The exact function of ANGPTL4 in ovarian carcinogenesis is unclear; it may be that the function of ANGPTL4 is dependent on the level of transcript present and the tumour type it is expressed in.

HER3 is a member of the epidermal growth factor receptor family [73] and has been linked to resistance to a number of therapeutics such as gefitinib in lung cancer [74] and paclitaxel in breast cancer [75]. Unexpectedly, we found HER3 expression to trend towards down-regulation in partial responders and non-responders to chemotherapy compared to responders. This was unusual, as high HER3 expression is usually linked to more aggressive tumour features such as metastasis [76] and reduced survival [77]. However, a recent study investigating the process of epithelial to mesenchymal transition (EMT) in ovarian cancer cell lines found low HER3 expression in intermediate mesenchymal cells, cells which had a more aggressive phenotype due to resistance to anoikis (a form of programmed cell death) and increased spheroid-forming capability *in vitro* [78]; hypoxia has been shown to induce EMT in ovarian cancer cells [22]. It may be that an unknown molecule is negatively regulating HER3 expression in our population, or that subclones of cells are responsible for the overall effect of differing HER3 expression. Indeed, it has been recognised that tumour sampling is very important in molecular analyses due to intra-tumour heterogeneity [79], and the regions sampled in our study may not have been representative of the whole tumour. Interestingly, low HER3 expression may identify patients who are suitable for alternate forms of treatment such as α-tocopherol ether-linked acetic acid (α-TEA) [80].

## Conclusions

Overall, these results show that the most important determining factor for development of resistance is the presence of hypoxia during the treatment period, not prior to treatment thus highlighting the potential importance of simultaneously reducing tumour hypoxia and treating with chemotherapy. This may have particular importance in patients with large tumours who receive neoadjuvant chemotherapy. A number of pathways are responsible for the resistance to cisplatin observed due to hypoxia, and that there are many candidate biomarkers of hypoxia which could be explored in the context of ovarian cancer. We have also provided an initial validation of selected hypoxia-associated biomarkers in ovarian tumour samples. It will be important to expand the study and to validate these results at the protein level in future studies in order to elucidate their true importance.

## Additional file

Additional file 1: Figure S1. Graphical representation of genetic changes in A2780 and A2780cis. Chromosomal location plots depicting location of differentially expressed genes in A2780cis compared to A2780 (A) and in response to hypoxia in A2780 (B) and A2780cis (C). Genes up-regulated are depicted in yellow, down-regulated in red and unchanged in white. Heat maps displaying patterns of differential gene expression in A2780cis compared to A2780 (D), and in response to hypoxia in A2780 (E) and A2780cis (F). Up-regulated genes are depicted in yellow, and down-regulated in red. *n* = 3.

## Abbreviations

α-TEA: alpha-tocopherol ether-lined acetic acid
ABC: ATP-binding cassette
AGCC: Affymetrix GeneChip Command Console
ANGPTL4: Angiopoietin-like protein 4
BCA: Bicinchoninic acid
CA: Carbonic anhydrase
CAV: Caveolin
CD55: Complement decay accelerating factor
CHK: Checkpoint kinase
DAVID: Database for Annotation, Visualization and Integrated Discovery
DUSP: Dual specificity phosphatase
ECACC: European Collection of Cell Cultures
EMT: Epithelial to mesenchymal transition
FFPE: Formalin fixed paraffin embedded
GAPDH: Glyceraldehyde 3-phosphatase
GLUT: Glucose transporter
HER3: Human epidermal receptor 3
HIF: Hypoxia-inducible factor
HRE: Hypoxia regulated element
HRP: Horseradish peroxidise
IC_50_: Inhibitory concentration 50
iHOP: Information hyperlinked over proteins
Jak: Janus kinase
L1-CAM: L1-cell adhesion molecule
MAP: Mitogen activated protein
MTT: 3-(4,5-dimethylthiazol-2-yl)-2, 5-diphenyltetrazolium bromide
PAGE: Polyacrylamide gel electrophoresis
PAS: Per Arnt Sim
PBS: Phosphate buffered saline
PCR: Polymerase chain reaction
PDGF: Platelet derived growth factor
PMSF: Phenylmethylsulfonyl fluoride
RIN: Ribonucleic acid integrity number
RIPA: Radioimmunoprecipitation
RMA: Robust multiarray average
SDS: Sodium dodecyl sulphate
SFN: Stratifin
STAT3: Signal transducer and activator of transcription 3
