# Supplementary figures and images for: Identifying novel hypoxia-associated markers of chemoresistance in ovarian cancer
Source: BMC Cancer. 2015 Jul 25;15:547. doi: 10.1186/s12885-015-1539-8 (PMC4513971; doi:10.1186/s12885-015-1539-8)

**
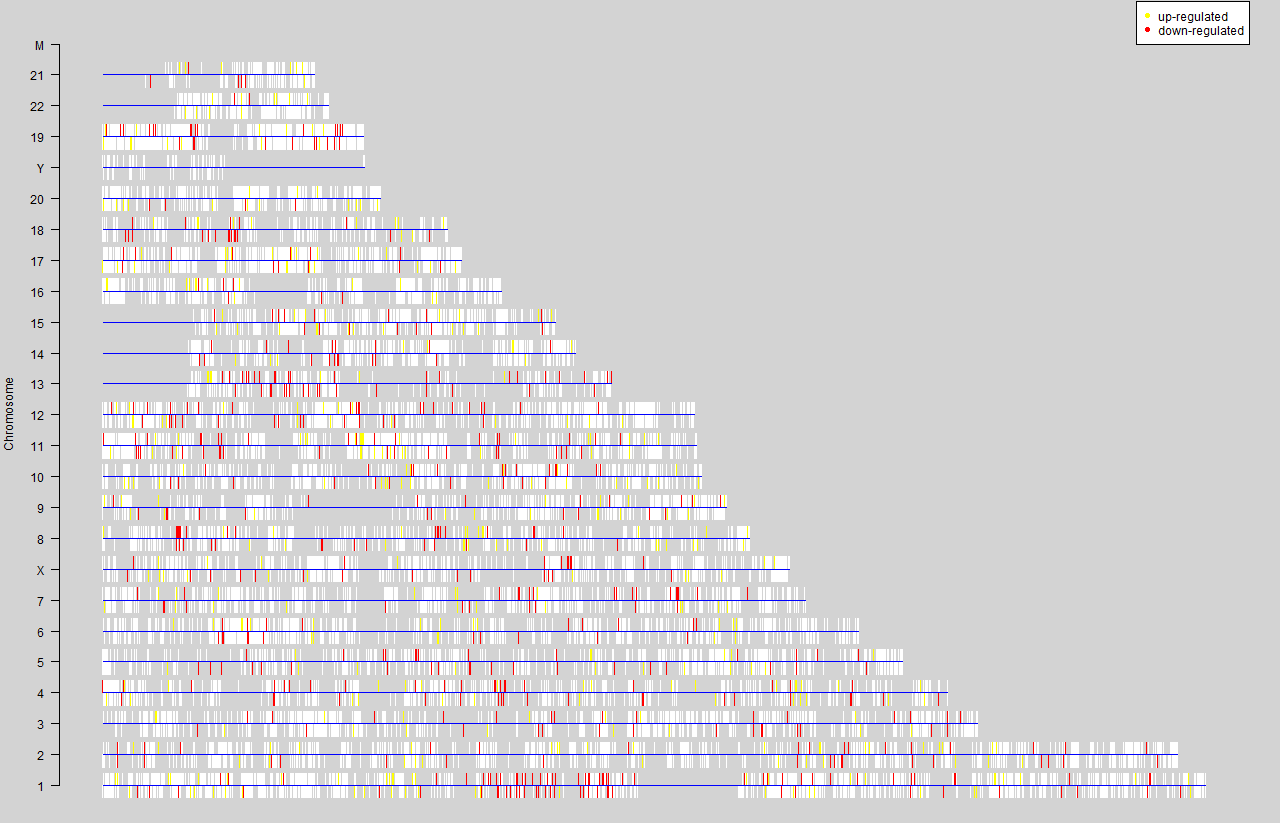

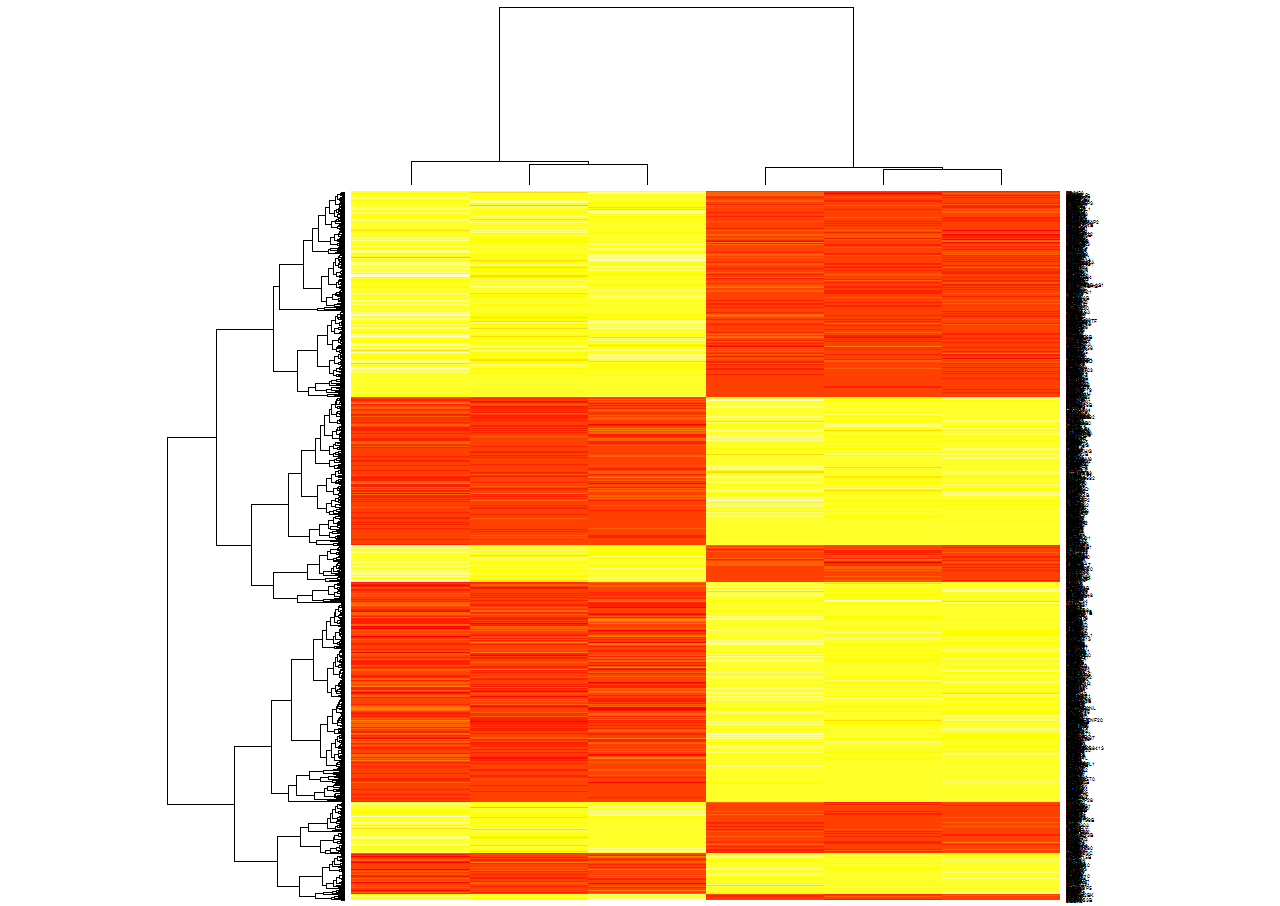

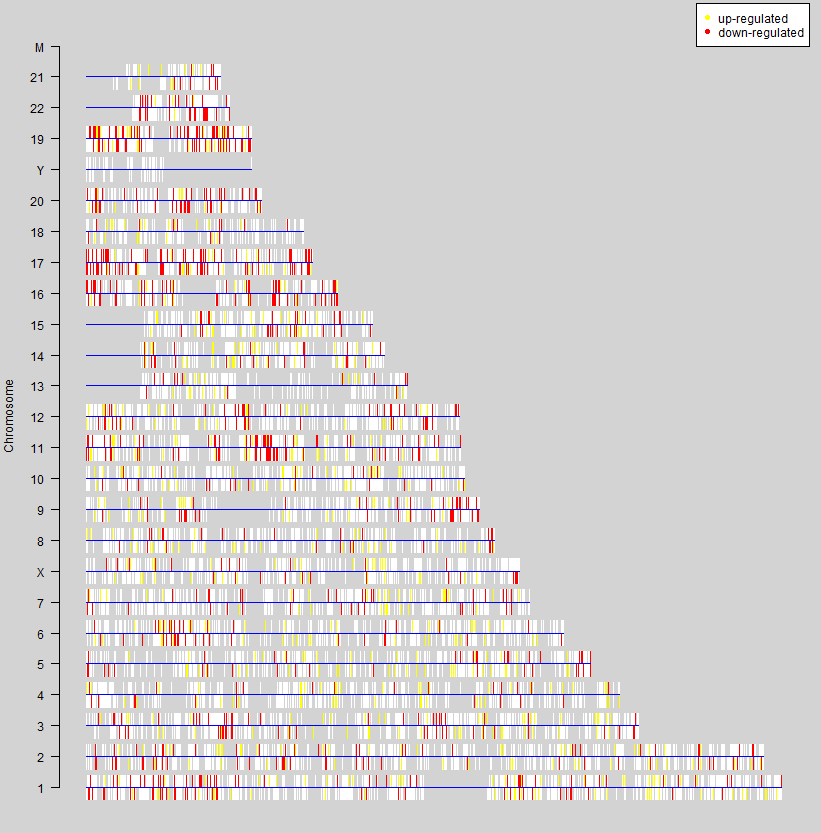

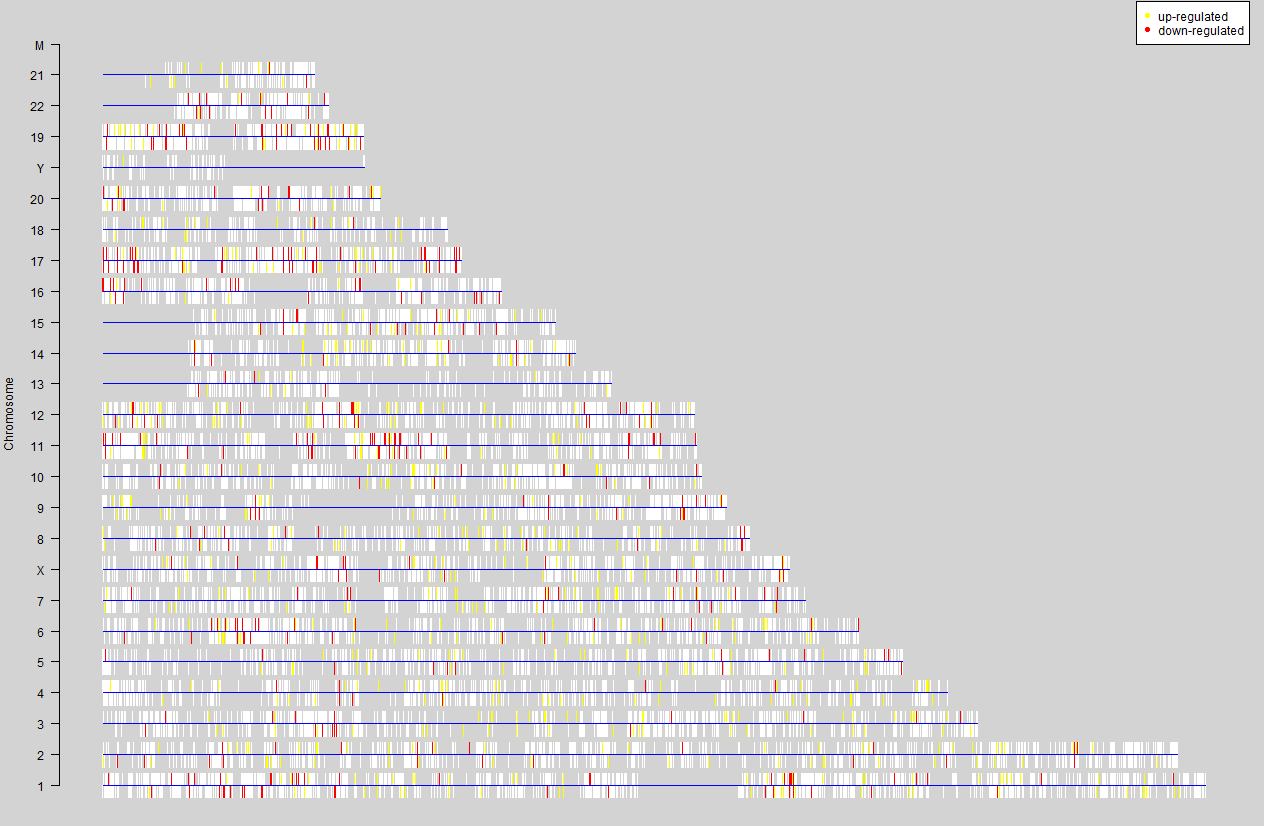

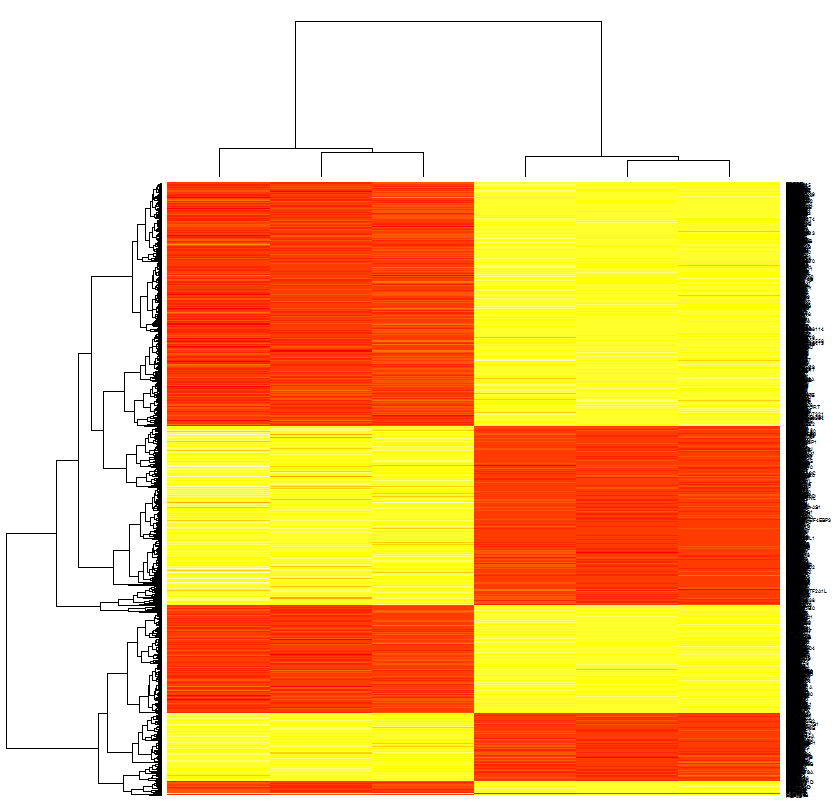

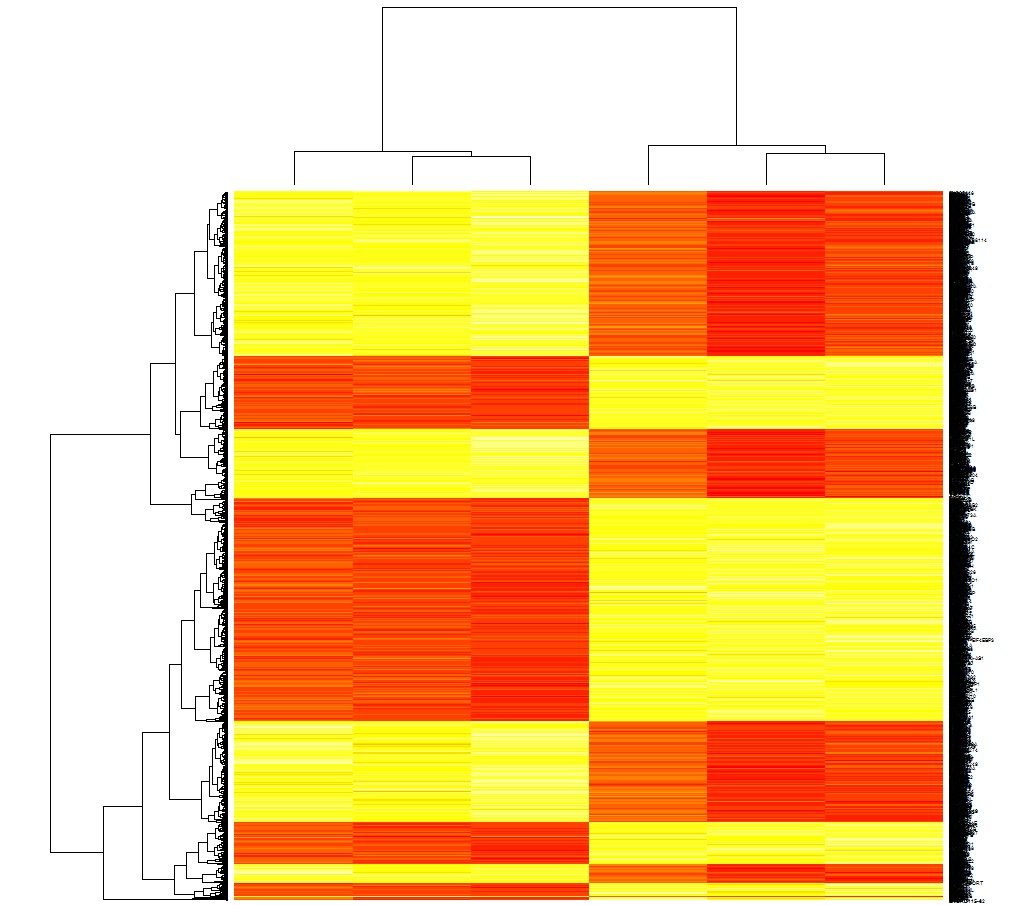
**

A

B

F

E

D

C

Supplement: Additional file 1: Figure S1. — Graphical representation of genetic changes in A2780 and A2780cis. Chromosomal location plots depicting location of differentially expressed genes in A2780cis compared to A2780 (A) and in response to hypoxia in A2780 (B) and A2780cis (C). Genes up-regulated are depicted in yellow, down-regulated in red and unchanged in white. Heat maps displaying patterns of differential gene expression in A2780cis compared to A2780 (D), and in response to hypoxia in A2780 (E) and A2780cis (F). Up-regulated genes are depicted in yellow, and down-regulated in red. n = 3. [file 12885_2015_1539_MOESM1_ESM.docx]
